# Improving access to the physical health clinic in a community first-episode psychosis service

**DOI:** 10.1192/bjo.2021.598

**Published:** 2021-06-18

**Authors:** Zena Tansley-Ahmed, Wei Han Lim

**Affiliations:** 1 Imperial College Healthcare NHS Trust; 2 Central and North West London NHS Foundation Trust

## Abstract

**Aims:**

Physical health outcomes are poor for patients with severe mental illness as demonstrated by the significant mortality gap present globally.[1] Access to and engagement with care is a key factor underpinning this disparity.[2] The Early Intervention in Psychosis service works with young people from 14-35 experiencing a first episode of psychosis in the community. Within the service, difficulties in engagement have been reflected in the high ‘no-show’ rates observed in the Foundation Year 2 trainee doctor-led physical health clinic. This quality improvement project aimed to reduce the ‘did not attend’ (DNA) rate in the physical health clinic by 20% in order to improve patient outcomes, particularly in the context of their physical health.

**Method:**

The project took place between September and November 2020, over the course of 10 weeks. A driver diagram was constructed to identify key influencing factors and subsequent change ideas. In order to implement each of these changes, three cycles within the Plan, Do, Study, Act (PDSA) ramp framework were completed. These consisted of phone reminders within 48 hours of appointments, a teaching session for staff alongside the distribution of an accompanying information leaflet and increased flexibility in clinic times with opportunistic appointments. The change ideas were cumulative with each cycle lasting a duration of seventeen days.

**Result:**

The baseline DNA rate was calculated based on the preceding month and found to be 55%. Following cycle one of the project, there was a significant reduction in DNA rates to 30% although this remained relatively stable at 33% after cycle two. By the end of cycle three when all interventions had been introduced, the DNA rate had dropped to 22%. As such, a total drop in DNA rate of over 30% was achieved which surpassed the initial aim of the project.

**Conclusion:**

The outcomes of this project demonstrate that the introduction of even simple measures can lead to positive change. Successful implementation of these changes requires teamwork and a culture of openness and flexibility. Feedback from team members, particularly care coordinators, also indicated better resulting engagement of clients with the service overall, suggesting potential for both improved mental and physical health outcomes. Next steps for this project may involve not only continued implementation of established changes but also service user input and scope for virtual consultations particularly in light of current COVID-19 restrictions.

